# Physical inactivity among physiotherapy undergraduates: exploring the knowledge-practice gap

**DOI:** 10.1186/s13102-016-0063-8

**Published:** 2016-12-07

**Authors:** Chathuranga Ranasinghe, Chathurani Sigera, Priyanga Ranasinghe, Ranil Jayawardena, Ayodya C. R. Ranasinghe, Andrew P. Hills, Neil King

**Affiliations:** 1 Department of Allied Health Sciences, Faculty of Medicine, University of Colombo, Colombo, Sri Lanka; 2 School of Exercise and Nutrition Sciences, Faculty of Health, Queensland University of Technology, Brisbane, Australia; 3 Ministry of Health and Nutrition, Colombo, Sri Lanka; 4 Department of Pharmacology, Faculty of Medicine, University of Colombo, Colombo, Sri Lanka; 5 Department of Physiology, Faculty of Medicine, University of Colombo, Colombo, Sri Lanka; 6 School of Health Sciences, University of Tasmania, Launceston, TAS Australia

**Keywords:** Physical activity, Physiotherapy undergraduates, Sri Lanka, Knowledge-practice gap

## Abstract

**Background:**

Physical inactivity is a common risk factor for several non-communicable diseases (NCDs). Increasing physical activity could reduce the burden of disease due to major NCDs and increase life expectancy. Undergraduate physiotherapy students represent a group of young-adults expected to have a good knowledge of physical activity. We evaluated physical activity levels of undergraduate physiotherapy students of University of Colombo, Sri Lanka and determined their motives and barriers for participation in physical activity.

**Methods:**

All physiotherapy undergraduates studying at the University of Colombo, Sri Lanka in 2013 were invited for the study. Phase one was a quantitative study to evaluate the physical activity levels and phase two was a qualitative study to identify motives and barriers for physical activity and sports in the same cohort. Physical activity levels (phase 1) were assessed using the interviewer administered International Physical Activity Questionnaire (long-version). The qualitative study (phase 2) was conducted in the same population using Focus Group Discussions (*n* = 3) and individual In-depth Interviews (*n* = 5).

**Results:**

Sample size in phase 1 and phase 2 were 113 (response rate = 98%; [N-115]) and 87 (response rat = 97%; [N-90]) respectively. Mean age (±SD) of participants was 23.4 ± 1 years. The mean weekly total MET minutes (±SD) of the study population was 1791.25 ± 3097. According to the IPAQ categorical score a higher percentage of participants were ‘inactive’ (48.7%), while only 15.9% were in the ‘Highly active’ group. Lack of support and encouragement received during childhood to engage in sports activity seem to have played an important role in continuing their exercise behavior through to the adult life. Academic activities were given priority by both parents and teachers. The environment and support from teachers, family and friends were important to initiate and adhere to sports and physical activity.

**Conclusions:**

A higher percentage of participants were ‘inactive’, in spite of belonging to a group which is presumed to be knowledgeable regarding the benefits of physical activity. A significant negative attitude towards physical activity was observed in this cohort of young-adults. This seems to stem from earlier in life, due to lack of support and motivation for physical exercise and sports, received during primary and secondary schooling. This negative attitude has become a significant ‘internal’ barrier, which has not been changed in spite of their education.

**Electronic supplementary material:**

The online version of this article (doi:10.1186/s13102-016-0063-8) contains supplementary material, which is available to authorized users.

## Background

Physical inactivity is a common risk factor for several non-communicable diseases (NCDs), including type 2 diabetes (T2DM) and cardiovascular disease [1]. The prevalence of NCDs has reached epidemic proportions, becoming the leading cause of death in adults in most countries globally. Worldwide, physical inactivity contributes to 6% of the burden of disease from coronary heart disease, 7% of T2DM, 10% of breast cancer and 10% of colon cancer [2]. Studies have shown that a reduction in physical inactivity could reduce the burden of disease due to major NCDs by 6-10% and increase life expectancy of the world’s population by 0.68 years [3]. Physical inactivity is associated with a considerable economic burden, accounting for 1.5–3.0% of total direct healthcare costs in developed countries [4]. Furthermore, engagement in habitual physical activity presents a protective effect against the onset of several NCDs, including cardiovascular disease and T2DM [3].

It is estimated that nearly one-fifth of the world’s population is physically inactive [5]. Promoting physical activity amongst adolescents and young-adults is a sensible strategy likely to help reduce physical inactivity levels and the associated disease burden in future generations. Health behaviors developed during adolescence and young adulthood tend to persist into adulthood [6]. However, recent studies have shown a decline in physical activity levels among young-adults [7]. This is more evident in European developed countries, where many teenagers and young-adults do not meet physical activity guidelines for health [8]. However, the situation in developing countries, including in the South Asian region, is not known. The development of effective interventions for the promotion of physical activity among teenagers and young-adults has become a recent priority in public health research [9].

South Asia, commonly known as the Indian sub-continent, is home to almost one-fifth of the world’s population and is comprised of many diverse ethnic, linguistic and religious groups. South Asia has a high disease burden due to diabetes and other NCDs associated with physical inactivity [10]. Furthermore, studies have shown that the majority of South Asian adults are physically inactive, especially during their leisure-time [11]. Evidence regarding physical activity among young-adults is limited. However it is important to understand barriers to engagement in physical activity among this population, including perceptions and knowledge about physical activity to develop effective evidence-based interventions. Qualitative studies can provide an in-depth insight into individuals’ experiences and perceptions of the motives and barriers for participation in sport and physical activity [12].

Undergraduate physiotherapy students represent a group of young-adults expected to have a good working knowledge of physical activity and related health benefits. Furthermore, graduate physiotherapists may be considered as important health advocates. They are expected to promote and prescribe exercise to patients and the general public. Presently there are no published data on physical activity levels in this group of young-adults. It is important to identify if there are any gaps between the knowledge and personal practice of physiotherapy students, particularly with respect to their motives and barriers for engagement in physical activity. The aim of the present study was to evaluate the physical activity levels of undergraduate physiotherapy students of the University of Colombo, Sri Lanka and determine their motives and barriers for participation in physical activity and exercise.

## Methods

### Study population and sampling

All physiotherapy undergraduates studying at the University of Colombo, Sri Lanka in 2013 were invited to participate in the study (n-115). The undergraduate Physiotherapy programme is a 4-year programme conducted in English, involving both male and female students aged 20–25 years. The study was conducted in 2 phases. Phase one was a quantitative study to evaluate the physical activity levels of physiotherapy students (June-August 2013) and phase two was a qualitative study to identify motives and barriers for physical activity and sports in the same cohort (August–November 2013). The participants were approached directly and informed written consent was obtained from study participants prior to data collection. Ethical approval was provided by the Ethics Review Committee, Faculty of Medicine, University of Colombo, Sri Lanka (EC-13-049).

### Data collection – quantitative study (phase 1)

Physical activity levels of undergraduate physiotherapy students were assessed using the interviewer administered (CR) International Physical Activity Questionnaire (IPAQ long version), which has shown to have acceptable validity when measuring physical activity in healthy adults [13]. Physical activity in the present study is expressed as categorical and continuous measurements as defined by the IPAQ. The continuous score estimates the weekly energy expenditure expressed in MET minutes/week (Metabolic Equivalent-Minutes) obtained by multiplying the value of energy expenditure for the given physical activity in METs by the weekly frequency (days per week) and the time in minutes (minutes per day). The categorical score classifies an individual into one of three categories: ‘Inactive’, ‘Moderately active’ and ‘Highly active’. The ‘Inactive’ category includes those who do not perform any physical activity or those reporting some activity, but not enough to meet other categories. ‘Moderately active’ refers to meeting either of the following criteria: a) 3 or more days of vigorous-intensity activity of at least 20 min per day; or b) 5 or more days of moderate-intensity activity and/or walking of at least 30 min per day; or c) 5 or more days of any combination of walking, moderate-intensity or vigorous intensity activities achieving a minimum total physical activity of at least 600 MET-minutes/week. Individuals who meet at least one of the above criteria would be defined as accumulating a minimum level of activity and therefore be classified as ‘Moderately active’. The two criteria for classification as ‘Highly active’ are: a) vigorous-intensity activity on at least 3 days achieving a minimum total physical activity of at least 1500 MET-minutes/week; or b) 7 or more days of any combination of walking, moderate-intensity or vigorous-intensity activities achieving a minimum total physical activity of at least 3000 MET-minutes/week. Activity patterns are determined in all physical activity domains (leisure, work, transport and household activities).

### Data collection – qualitative study (phase 2)

A qualitative study was conducted in the same study population using Focus Group Discussions (FGDs) and In-depth Interviews (IDIs). The FGDs/IDIs were conducted according to guidelines provided by previous studies and the details are reported according to the COREQ guidelines on Qualitative Research [14–16]. Initially, themes to be discussed in the FGDs and IDIs were derived using a questionnaire with two open-ended questions regarding the barriers for adherence and continuation of engagement in physical activity/sports (“*please mention five reasons for you to engage/not engage in regular leisure time physical activities and prioritize those reasons”).*.

Three FGDs (FGD_1_-FGD_3_) were conducted by a facilitator and included 8–10 participants (S_1_–S_10_). The composition of each group was decided according to gender, ethnicity and age and purposive selection achieved the maximum variation on the above parameters. The FGD was conducted at the Department of Allied Health Sciences, using a set of open-ended semi-structured questions to guide the participants and to keep uniformity between the different focus groups (Additional file 1). The duration of each FGD was one hour, and each FGD was audio tape recorded after obtaining consent from the participants. Two independent observers at each FGD (CS and ACRR) were responsible for verbatim recording/transcribing and documenting emotional responses, respectively and they were responsible for making field notes during the FGD. The facilitator/moderator was a male health care practitioner and a trained researcher, with experience in conducting focus group discussions (CR, MBBS, Lecturer). The facilitator provided guidance, maintained focus, stimulated constructive debate, regulated the flow of discussion and ensured time adherence, while maintaining a neutral stance on contents of discussion. Data collection was stopped when data saturation was reached. To determine when data saturation occurs, analysis was conducted concurrently with data collection (as described below), and when no new information relevant to each theme was generated from two subsequent FGDs the data collection was stopped. After completing the FGDs, at least one student from each group was recruited for in-depth interviews (*n* = 5). We identified one participant from each group who was not comfortable talking openly in the FGD. The IDIs were used to further explore and clarify more in depth their views of the opinions discussed in FGDs.

### Data analysis

Quantitative data were analyzed using Statistical Package for Social Sciences (SPSS) software, version 14. Descriptive data was presented as percentages or as mean ± standard deviations. Significance of associations was tested using Chi square for categorical variables and Student’s *t*-test for continuous variables. Directed content analysis of qualitative data was conducted with the assistance of NVIVO v10.0 (QSR International, Southport, UK). The topics/themes were selected prior to the analysis and participants’ responses were grouped under these topics/themes. The facilitator and two observers were entrusted with the task of providing an analysis of verbal and non-verbal responses of participants in each FGD within twenty-four hours of conclusion. The facilitator and two observers analyzed their respective FGD using their field notes and the audio tape-recorded data collectively immediately after the conclusion of the FGD. The resulting transcripts were returned to participants for comment and/or correction. Each FGD produced one complete document, and all documents were collectively analyzed by the research team. A similar method was used for the analysis of the data emerging from the IDIs.

## Results

### Socio-demographic characteristics

All students enrolled in the Physiotherapy programme were eligible to participate in Phase 1 of the study (*n* = 113, response rate = 98%). Mean age of participants was 23.4 ± 1 years (range 20–25 years), and 32.8% were males. For the qualitative study (Phase 2) only students in years 1–3 were included as final year students had academic commitments (*n* = 87, response rate = 97%). Mean age was 22.8 ± 1.1 years (range 20–24 years), and 41.4% were males. The majority of the students in the overall study were from rural areas (55.0%) and Sinhalese (85.2%) in ethnicity.

### Phase 1 – physical activity

The mean weekly total MET minutes of the study population was 1791.25 ± 3097 (males 1982.5 ± 3097 and females 1695.63 ± 2676, p-NS). According to the IPAQ categorical score a significant percentage of participants were ‘inactive’, while only 15.9% were in the ‘Highly active’ group (Table 1). Although as described above many of students achieved the MET-minutes threshold for ‘Moderate activity’, it was not achieved on at least 5 days of the week, resulting in majority being classified as ‘inactive’. In the specific domains, physical inactivity was higher during ‘leisure-time’ compared to other domains, with most being relatively active in the transport and household chores domains. Females were more active than males in other domains except the leisure-time activity domain (Table 1).

**Table 1:** Domain specific activity levels

Domains	Activity category	Prevalence		
		All (*n* = 113)	Males (*n* = 37)	Females (*n* = 76)
Overall	Inactive	48.7%	51.3%	47.4%
	Moderately active	35.4%	35.2%	35.5%
	Highly active	15.9%	13.5%	17.1%
Leisure time activity	Inactive	80.6%	70.3%	85.5%
	Moderately active	15.0%	24.3%	10.6%
	Highly active	4.4%	5.4%	3.9%
Work/job based activity	Inactive	66.4%	70.3%	64.5%
	Moderately active	33.6%	29.7%	35.5%
	Highly active	0	0	0
Transport related activity	Inactive	44.2%	45.9%	43.4%
	Moderately active	47.8%	40.5%	51.3%
	Highly active	8.0%	13.6%	5.3%
House hold/garden choirs	Inactive	57.5%	59.5%	56.6%
	Moderately active	33.7%	27.0%	36.8%
	Highly active	8.8%	13.5%	6.6%

### Phase 2 - qualitative study

The following four themes were identified in response to the open-ended written questionnaires regarding barriers to engage in physical activity; time, motivation and support, facilities and personal reasons (in order of priority expressed). The reasons expressed by the study participants in response to the questionnaire are summarized in Table 2. These prioritized reasons (barriers) expressed by the participants were used to develop interviewer guidelines for the FGDs and IDIs and the information derived from the FGDs and IDIs were analyzed according to the above five themes (directed content analysis).

**Table 2:** Barriers to engage in sports and physical activity

Themes	Reasons
Time	*“Increased academic work load reduces the time to engage in sports”* *“Lack of appropriate time management to include exercise into daily routine”* *“More time spent on travelling”* *“Have other daily activities after going home to engage in”*
Motivation & Support	*“Do not like sports. Like to be calm and alone watch TV, browse internet or read”* *“Do not like to get hurt/feel pain”* *“Get tired quickly”* *“Not enjoyable”* *“Increase workload increases stress, preventing involvement in sports”* *“Nobody appreciates doing sports (peers, teachers)”* *“Need a partner or a friend to do sports”* *“No support from family, school and teachers”* *“Need a supervisor or a coach”* *“Do not like to get tired. Can’t study after exercising”*
Facilities	*“Do not have a gymnasium at place of study”* *“At hostel there is no room or appropriate space to exercise”* *“There is no ground nearby, must travel by bus”* *“No money to go to a gym or buy equipment”*
Personal	*“Do not know benefits”* *“Medical reasons (wheezing)”* *“Do not like to being in the sun”* *“Lack of nutritious food in hostels and boarding places. Do not have enough energy”* *“Difficult to change dresses”*

### Theme 1: time

Lack of time was the most prioritized barrier for engagement in physical activity during the initial quantitative screening. However, this was less prominent by the participants during the FGDs and the IDIs and many agreed that it is a problem of managing one’s own time effectively.

*“We mention time only to justify ourselves. If there is an attitude of doing sport, any way you can change the time and the schedule”. (P3: Male, 24 years)*

Some students living in hostels mentioned that travelling and increased academic workload reduced their ability to initiate or maintain any sports or other physical activity.

*“When we go to the hostel we are tired. We don’t have good food like at home. We once started jogging in the morning for some time, then stopped. When we run and come it’s 7.30 am. We have to go for lectures at 8.00 am. There is not enough time.”(P6: Male, 22 years)*

### Theme 2: support and motivation

Support and encouragement received during childhood to engage in sports activity seem to have played an important role in continuing their exercise behavior through to the adult life.

*“When I was schooling I did not do many sports. That might also be a reason not being active now. It might be the mentality carried from my childhood and may be the environment I was in at that time.” (P15: Female, 24 years)*

The environment and support from teachers, family and friends were important to initiate and adhere to sports and physical activity. During childhood teachers and parents did not motivate some of them. Academic activities were given priority by both parents and teachers.

*“My family asked me to go to the campus as it will give me a job and playing will not. Teachers also didn’t tell about the benefits of sports or move us towards them” (P8: Male, 23 years)*

*“A well respected teacher once told me not to do extracurricular things, but to study.” (P21: Female, 22 years)*

As undergraduates, they also expressed a lack of support from teachers and mentioned that the Institute (university curriculum and structure) has not promoted or motivated them to do sports or physical activity.

*“People who came for interfaculty games say that they won’t come next time. When asked why, they said that academic performance has gone down, got repeated in exams and demoted to the junior batch. Even faculty should think about this, that these students do sports for the university. So must be able to reschedule the exams. The students think that I do this for the university and university doesn’t do anything back for me” (P3: Male, 24 years)*

*“Most of my teachers, parents and others motivated to get education and go to the university. So most our target was to go to a university and get a degree which will help to get a job and settle in life. Teachers told us ‘Don’t do sports! Concentrate on education!’ They did not encouraged us to do sports” (P17: Male, 23 years)*

Peer support and involvement in peer groups seem to be an important motivational factor to adhere to sports and physical activity. Most of the females expressed the need to have a partner or a friend of same gender during activity.

*“I don’t feel like going alone to the ground and play. Feel shy, if there is crowd I would like. If alone I won’t go” (P2: Female, 22 years)*

*“When I was schooling I played netball. Now when going to grounds if girls are not going, we also don’t. The environment and friends matter.”(P11: Female, 21 years)*

Some of the Males also expressed the importance of having a partner,

*“Better to have a partner. At my boarding place there was nobody and I was living in an unknown area. I didn’t go for exercise alone*.” (*P3: Male, 24 years)*

Knowledge regarding physical activity gained from their course and commitments as physiotherapists in the future was mentioned as motivators by the students.

*“There is some influence from the course I do. The knowledge I got. If I do not exercise that I will be getting diseases.”(P9: Female, 25 years)*

*“Now we have a motivation because we are doing a health related course. But if we did it as a subject from childhood would have been better!!! We have to give emphasis that these kind of things to happen in policy” (P3: Male, 24 years)*

*“We being physiotherapists, can’t tell others to exercise without us doing them. There is a motivation from our profession to exercise.” (P7: Male, 23 years)*

### Theme 3: facilities

Limited facilities and lack of accessibility to engage in sports and physical activity was expressed by some participants, for example lectures were distant to the main playground and gymnasium and the need to travel had resulted in reduced participation in sports and recreational activities.

*“Mainly because the ground was distant, number of friends going to the ground reduced. Also our unit premises do not have enough space to do indoor or outdoor activity. Previously we got together and played badminton. Now there is no space for that even. We only can play carom.” (P4: Male, 24 years)*

Students have tried to establish a gym from their own funds via the Student Union, but they have been faced with problems maintaining it.

*“Facilities are a problem at the unit. We established a Gym. Basic things are there but no facilities to wash, only strengthening activities are available there no aerobics part. It’s not a barrier but it is a problem.”(P3: Male, 24 years)*

### Theme 4: Personal

For some participants, low self-efficacy was causing them to not engage in sports and physical activity.

*“When I was very young I tried sports but I am not good at it. When we are not good at something we do not perform. So subsequently you detach from it. So after that I did not try to get involve in sports. Because I have no ability in that. I tried to improve something that I am good at.” (P9: Female, 25 years)*

The group of students studied here was a mix of different ethnic religious groups from diverse cultures. Cultural barriers affected exercise behavior in certain religions, for example Islam and especially in females not being encouraged to engage in sports after a certain age.

*“When I was very young teachers parents encouraged me for sports. After grade 9 all of them did not like that. In my school no girl did sports. So they look at us differently if we did sports. Girls go for marching and drills. Running is a sport that is not appropriate for our dress. My family members also did not like that. You have to dress suiting for exercise like a t-shirt. We don’t wear t shirts. I do activities in the hostel. Can’t do that in the campus due to the culture problem.” (P5: Female, 23 years)*

Many females expressed that their perception about physical activity and sports changed with time, ‘females being less active and males being active and playful’. Females of all cultural backgrounds agreed on this.

*“I think according to gender physical activity differs. I don’t know. We all play together in childhood. But when we grow older that change, girls do not engage in playing much, it reduces. I don’t know the reason, must be the culture we are in” (P12: Female, 23 years)*

## Discussion

Physical activity has many beneficial physical and mental health effects. Physical inactivity is considered the fourth leading risk factor for global mortality [2]. A recent systematic review highlighted that the majority of South Asians are physically inactive, especially during leisure-time [17]. Our results support these findings, however it is particularly alarming as the current cohort could be considered unique and expected to be active, as they are constantly exposed to the importance of physical activity in their academic curriculum. Furthermore, participants are also trained in the prescription, education and motivation of their patients to adopt physical exercise regimes. Hence, our results indicate a clear mismatch between the knowledge and practice in this group of young-adults. In addition, similar studies among physiotherapy students and physiotherapist conducted in developed countries such as Australia and Latvia, has indicated that majority of the participants were either ‘Moderately’ or ‘Highly’ active [18, 19].

A number of studies have shown that health care workers exhibit the same unhealthy behaviors as the general population [20]. Similarly, evidence also demonstrates that the personal health behaviors and attitudes of health professionals may influence how they practice clinically and may also influence how patients view their credibility as health promoters [20]. Hence, it is important to explore the reasons for the observed discrepancy between knowledge and practice in this group using qualitative methods. The barriers for physical activity in the general population of South Asian young-adults are likely to be similar to those observed in this group. Our results demonstrate that insufficient time, lack of motivation and support, inadequate facilities and other personal reasons were the primary contributing factors towards physical inactivity in this group.

Perceived lack of time is a well-known barrier to engagement in physical activity, especially among young-adults due to occupational and educational commitments [21]. Similarly, lack of facilities is another commonly identified barrier [22]. Sallis et al. found that, after control for individual characteristics, closer proximity and higher density of exercise facilities were significantly associated with increased frequency of exercise [23]. An environmental intervention aimed at reducing barriers to physical activity (including increasing the availability of physical activity–related equipment and facilities) revealed statistically significant positive changes in overall fitness measures within the intervention community [24]. Hence, it is evident that improving facilities and environmental related factors are important to encourage physical activity.

However, all these ‘external’ barriers can be overcome with the correct attitude and motivation. The most important finding from the present study is the significant negative attitude towards physical activity expressed by study participants. This attitude appears to stem from earlier in life due to lack of support and motivation for physical exercise and sports, received during primary and secondary schooling. Many expressed they were more focused on academic work during childhood and adolescence acting on guidance received from their parents and teachers. Academic performance during secondary education in the Sri Lankan context is very important for a stable career and future income. As is the case elsewhere, it is difficult to have a career based solely on sport. Less than 10% of students are eligible to enter state universities due to the limited number of university places available, leading to a high level of academic competitiveness. Accordingly, most teachers and parents promote academic achievement over sports. Even at times when teachers have promoted sports activities, students themselves felt that academic performance is affected by involving in sports. Planning the future financial stability or job, based on academic performances have affected their exercise behavior in childhood and transmitted to their adult life.

In this cohort we also observed that low-self efficacy was another factor for not pursuing physical activity. Self-efficacy is defined as one's beliefs in their own capabilities to successfully accomplish a specific task. Self-efficacy has been demonstrated to be particularly influential in the adoption of physical activity [25]. There appears to be greater opportunity to mediate behavior through cognitive control than when behavior has become more habitual [26]. The role played by self-efficacy in physical activity behavior appears to be quite consistent across ages and populations [27]. Higher levels of self-efficacy is known to be associated with participation in more physical activity [2]. Those with high self-efficacy for walking, stronger intentions to be active, and a plan for activity were twice as likely to meet the physical activity recommendations [28]. The perceived low-self efficacy in the present cohort can become a significant barrier for initiation and maintenance of physical activity. Improving self-efficacy is known to be an effective method increase physical activity. Evidence from some trials supports the view that incorporating the theory of self-efficacy into the design of an exercise intervention is beneficial [29]. Hence, self-efficacy is an important factor that needs to be considered when planning physical activity interventions.

These negative attitudes have become a significant ‘internal’ barriers, which has not been changed in spite of the education received about the importance of physical activity. This is likely to be an issue not only in Sri Lanka but also in other South Asian countries that share similar socio-cultural values and beliefs. This negative attitude also persists in spite of evidence suggesting a positive relationship between physical activity/exercise/sports and academic performance [17]. Interventions aimed at enhancing physical activity in young-adults are less likely to be successful without such ‘internal’ barriers being addressed, ideally much earlier during early childhood and adolescence. Better outcomes are likely by changing the attitudes and beliefs of parents and teachers through carefully planned educational programmes and other interventions. In future studies it would be valuable to compare data from this cohort with non-health undergraduate students. Furthermore, university policies should change to encourage exercise and sports among undergraduates and providing facilities close to university premises.

It is evident from our findings that psychosocial constructs needs to be given careful consideration when planning interventions to promote physical activity. Although environmental factors can be corrected within a short time period of time, it is likely that many years are required to change certain ‘internal’ barriers and attitudes in younger generations. The socio-ecological approach emphasizes that health promotion should focus not only on intrapersonal behavioral factors but also on the multiple-level factors that influence the specific behavior in question [30]. The socio-ecological model thus focuses on the interrelationships between individuals and the social, physical and policy environment [31]. The model helps us to identify opportunities to promote physical activity by recognizing the individual (eg sex, beliefs and attitudes), behavioral (sedentary and active time), social environmental (family, teachers, peers) and physical environmental (eg availability of equipment and facilities) factors that may influence one’s ability to be sufficiently physically active [32]. The results from the present study recognizes such individual level and physical environmental factors that influences physical activity. Hence there is a need for using comprehensive interventional models addressing multiple factors when planning health promotional activities in the present population.

This study helped to fill a recognized research gap in the region and identified a key primary ‘internal’ barrier to physical activity among young-adults. The qualitative methods used were rigorous and the study suggested some new findings regarding perceptions on physical activity. There are several limitations in the present study. The majority of respondents were Sinhalese (85.2%) in ethnicity. Studies have shown that Sri Lankan Tamils generally have a higher level of physical activity compared to other ethnic groups [33]. Furthermore, physical activity in the present study was self-reported using the IPAQ questionnaire which typically over-estimates physical activity [34, 35]. Despite this, self-report remains the simplest, most feasible and affordable instrument for physical activity surveillance with the IPAQ instrument previously validated for use among Sri Lankan adults.

## Conclusions

In this group of young-adults the observed physical activity levels were low, with a majority being physically inactive. A significant negative attitude towards physical activity was observed in a cohort of young-adults with good knowledge about its benefits. This seems to stem from earlier in life, due to lack of support and motivation for physical exercise and sports, received during primary and secondary schooling. This negative attitude has become a significant ‘internal’ barrier, which has not been changed in spite of their education.

## Additional file

Additional file 1: Interviewer Guide (contains the set of open-ended semi-structured questions used during the focus group discussions by the interviewers to guide the participants and to keep uniformity between the different focus groups). (DOC 31 kb)

## Abbreviations

FGD: Focus Group Discussion
IDI: In-depth interview
IPAQ: International physical activity questionnaire
MET: Metabolic equivalents
NCD: Non-communicable disease
T2DM: Type 2 diabetes

## References

[1] Global Strategy on Diet, Physical Activity and Health [http://www.who.int/dietphysicalactivity/factsheet_adults/en/index.html]. Accessed 7 Nov 2016.

[2] Lee IM, Shiroma EJ, Lobelo F, Puska P, Blair SN, Katzmarzyk PT (2012). Effect of physical inactivity on major non-communicable diseases worldwide: an analysis of burden of disease and life expectancy. Lancet.

[3] Fernandes RA, Zanesco A (2010). Early physical activity promotes lower prevalence of chronic diseases in adulthood. Hypertens Res.

[4] Oldridge NB (2008). Economic burden of physical inactivity: healthcare costs associated with cardiovascular disease. Eur J Cardiovasc Prev Rehabil.

[5] Dumith SC, Hallal PC, Reis RS, Kohl HW (2011). Worldwide prevalence of physical inactivity and its association with human development index in 76 countries. Prev Med.

[6] Telama R, Yang X, Viikari J, Välimäki I, Wanne O, Raitakari O (2005). Physical activity from childhood to adulthood. Am J Prev Med.

[7] Van Mechellen W, Twisk J, Post G, Snel J, Kemper H (2000). Physical activity of young people: the Amsterdam Longitudinal Growth and Health Study. Med Sci Sports Exerc.

[8] Cavill N, Kahlmeier S, Racioppi F. Physical activity and health in Europe evidence for action. Denmark: World Health Organization; 2006.

[9] De Meester F, van Lenthe FJ, Spittaels H, Lien N, De Bourdeaudhuij I (2009). Interventions for promoting physical activity among European teenagers: a systematic review. Int J Behav Nutr Phys Act.

[10] Jayawardena R, Ranasinghe P, Byrne NM, Soares MJ, Katulanda P, Hills AP (2012). Prevalence and trends of the diabetes epidemic in South Asia: a systematic review and meta-analysis. BMC Public Health.

[11] Ranasinghe CD, Ranasinghe P, Jayawardena R, Misra A (2013). Physical activity patterns among South-Asian adults: a systematic review. Int J Behav Nutr Phys Act.

[12] Dixon-Woods M, Fitzpatrick R (2001). Qualitative research in systematic reviews. Has established a place for itself. BMJ.

[13] Hagstromer M, Oja P, Sjostrom M (2006). The International Physical Activity Questionnaire (IPAQ): a study of concurrent and construct validity. Public Health Nutr.

[14] Tong A, Sainsbury P, Craig J (2007). Consolidated criteria for reporting qualitative research (COREQ): a 32-item checklist for interviews and focus groups. Int J Qual Health Care.

[15] Wong LP (2008). Focus group discussion: a tool for health and medical research. Singapore Med J.

[16] Lakshman M, Charles M, Biswas M, Sinha L, Arora NK (2000). Focus group discussions in medical research. Indian J Pediatr.

[17] Morales J, Pellicer-Chenoll M, Garcia-Masso X, Gomis M, Gonzalez LM (2011). Relation between physical activity and academic performance in 3rd-year secondary education students. Percept Mot Skills.

[18] McPhail SM, Waite MC (2014). Physical activity and health-related quality of life among physiotherapists: a cross sectional survey in an Australian hospital and health service. J Occup Med Toxicol.

[19] Mihailova A, Kaminska I, Bernane A (2014). Physical activity in physiotherapy and physical education high school students. SHS web of conferences.

[20] While AE (2015). Promoting healthy behaviours - do we need to practice what we preach?. Lond J Prim care.

[21] Arzu D, Tuzun EH, Eker L (2006). Perceived barriers to physical activity in university students. J Sports Sci Med.

[22] Samara A, Nistrup A, Al-Rammah TY, Aro AR (2015). Lack of facilities rather than sociocultural factors as the primary barrier to physical activity among female Saudi university students. Int J Women’s Health.

[23] Sallis JF, Hovell MF, Hofstetter CR, Elder JP, Hackley M, Caspersen CJ, Powell KE (1990). Distance between homes and exercise facilities related to frequency of exercise among San Diego residents. Public Health Rep (Washington, DC : 1974).

[24] Linenger JM, Chesson CV, Nice DS (1991). Physical fitness gains following simple environmental change. Am J Prev Med.

[25] McAuley E, Blissmer B (2000). Self-efficacy determinants and consequences of physical activity. Exerc Sport Sci Rev.

[26] McAuley E, Jerome GJ, Marquez DX, Elavsky S, Blissmer B (2003). Exercise self-efficacy in older adults: social, affective, and behavioral influences. Ann Behav Med.

[27] De Bourdeaudhuij I, Sallis J (2002). Relative contribution of psychosocial variables to the explanation of physical activity in three population-based adult samples. Prev Med.

[28] Cleland VJ, Ball K, Salmon J, Timperio AF, Crawford DA (2010). Personal, social and environmental correlates of resilience to physical inactivity among women from socio-economically disadvantaged backgrounds. Health Educ Res.

[29] Rajati F, Sadeghi M, Feizi A, Sharifirad G, Hasandokht T, Mostafavi F (2014). Self-efficacy strategies to improve exercise in patients with heart failure: A systematic review. ARYA Atheroscler.

[30] Mehtala MA, Saakslahti AK, Inkinen ME, Poskiparta ME (2014). A socio-ecological approach to physical activity interventions in childcare: a systematic review. Int J Behav Nutr Phys Act.

[31] Stokols D (1996). Translating social ecological theory into guidelines for community health promotion. Am J Health Promot.

[32] Richard L, Potvin L, Kishchuk N, Prlic H, Green LW (1996). Assessment of the integration of the ecological approach in health promotion programs. Am J Health Promot.

[33] Qin L, Corpeleijn E, Jiang C, Thomas GN, Schooling CM, Zhang W, Cheng KK, Leung GM, Stolk RP, Lam TH (2010). Physical activity, adiposity, and diabetes risk in middle-aged and older Chinese population: the Guangzhou Biobank Cohort Study. Diabetes Care.

[34] Rzewnicki R, Vanden Auweele Y, De Bourdeaudhuij I (2003). Addressing overreporting on the International Physical Activity Questionnaire (IPAQ) telephone survey with a population sample. Public Health Nutr.

[35] Ainsworth BE, Macera CA, Jones DA, Reis JP, Addy CL, Bowles HR, Kohl HW (2006). Comparison of the 2001 BRFSS and the IPAQ physical activity questionnaires. Med Sci Sports Exerc.

