# Supplementary material for: Physical inactivity among physiotherapy undergraduates: exploring the knowledge-practice gap
Source: BMC Sports Sci Med Rehabil. 2016 Dec 7;8:39. doi: 10.1186/s13102-016-0063-8 (PMC5142393; doi:10.1186/s13102-016-0063-8)
Supplement: Additional file 1: — Interviewer Guide (contains the set of open-ended semi-structured questions used during the focus group discussions by the interviewers to guide the participants and to keep uniformity between the different focus groups). (DOC 31 kb) [file 13102_2016_63_MOESM1_ESM.doc]

**Perception of motives and barriers to participation in Physical activity among the undergraduates of BSc. Physiotherapy Faculty of Medicine, Colombo**

**Guide to Focus Group Discussion**

**Introduction**

Thank you for providing us your valuable time by participating in this discussion. We will be discussing today about knowledge, perception and barriers of physical activity and sports participation among physiotherapy undergraduates for healthy living. Understanding these barriers will be useful to implement appropriate public health interventions for you and other young adults.

We want to hear your opinions, and remember that there are no right or wrong answers. You are free to answer to the questions in any way you feel comfortable. You can refuse answering if you do not want to answer at all. If there is any unclear question do not hesitate to clarify and make us to explain more on it. We are planning to keep a tape record of this conversation to enable us to clarify unclear areas of this discussion later; we hope that you will consent to this. We guarantee that what is said will be kept strictly confidential.

Be comfortable – we hope you will find the session interesting and enjoyable.

**Question 1: What do you understand by a being active?**

Probes

- What is being physically active? What is exercising?
- Are you active? Inactive?
- Recommendations of physical activity for healthy living?
- Do you think is beneficial/ non beneficial to be active and why? (Health related, social, economical etc
- Do you think it is not beneficial to be active and why? (getting tired, getting thin, waste of time)

**Question 2: Why do you think you are not participating in physical activity/exercise/sports in your leisure time?**

Probes

- Lack of time?(why time not enough, what is your daily routine like)
- Lack of facilities? - reasons, suggestions
- Lack of Motivation?-What motivates you ,lack of energy , studies, culture
- Do you think support is important –(friends, family, society, institution)
- Any Personal reasons Other (do not want to get tired, hurt, pain, sun, figur**e,** just don’t like

**Question 3: What do you think about present health promotion of physical activity and sports participation for your age group/young adults?**

Probes

- By School
- Society
- Media
- Health sector
- Personalities used and themes used

**Question 4: How do you think we can improve physical activity of the public?**

Probes

- In the workplace, study set up
- During transport
- At home, during leisure time
